# Role of Mitochondria in the Oxidative Stress Induced by Electromagnetic Fields: Focus on Reproductive Systems

**DOI:** 10.1155/2018/5076271

**Published:** 2018-11-08

**Authors:** Silvano Junior Santini, Valeria Cordone, Stefano Falone, Mahmut Mijit, Carla Tatone, Fernanda Amicarelli, Giovanna Di Emidio

**Affiliations:** ^1^Dept. of Life, Health and Environmental Sciences, University of L'Aquila, 67100 L'Aquila, Italy; ^2^Infertility Service, San Salvatore Hospital, L'Aquila, 67100 L'Aquila, Italy; ^3^Institute of Translational Pharmacology (IFT), CNR, 67100 L'Aquila, Italy

## Abstract

Modern technologies relying on wireless communication systems have brought increasing levels of electromagnetic field (EMF) exposure. This increased research interest in the effects of these radiations on human health. There is compelling evidence that EMFs affect cell physiology by altering redox-related processes. Considering the importance of redox *milieu* in the biological competence of oocyte and sperm, we reviewed the existing literature regarding the effects of EMFs on reproductive systems. Given the role of mitochondria as the main source of reactive oxygen species (ROS), we focused on the hypothesis of a mitochondrial basis of EMF-induced reproductive toxicity. MEDLINE, Web of Science, and Scopus database were examined for peer-reviewed original articles by searching for the following keywords: “extremely low frequency electromagnetic fields (ELF-EMFs),” “radiofrequency (RF),” “microwaves,” “Wi-Fi,” “mobile phone,” “oxidative stress,” “mitochondria,” “fertility,” “sperm,” “testis,” “oocyte,” “ovarian follicle,” and “embryo.” These keywords were combined with other search phrases relevant to the topic. Although we reported contradictory data due to lack of uniformity in the experimental designs, a growing body of evidence suggests that EMF exposure during spermatogenesis induces increased ROS production associated with decreased ROS scavenging activity. Numerous studies revealed the detrimental effects of EMFs from mobile phones, laptops, and other electric devices on sperm quality and provide evidence for extensive electron leakage from the mitochondrial electron transport chain as the main cause of EMF damage. In female reproductive systems, the contribution of oxidative stress to EMF-induced damages and the evidence of mitochondrial origin of ROS overproduction are reported, as well. In conclusion, mitochondria seem to play an important role as source of ROS in both male and female reproductive systems under EMF exposure. Future and more standardized studies are required for a better understanding of molecular mechanisms underlying EMF potential challenge to our reproductive system in order to improve preventive strategies.

## 1. Introduction

Power transmission and electrical equipment have changed our lives. In addition, modern technologies relying on wireless communication systems are among the fastest-growing industries worldwide. Since 1986 when the first study investigated the impact of electric blankets and heated water on fertility potential [1], there has been an increasing interest in studying the effects of exposure to nonionizing electromagnetic radiations on reproductive functions. A significant decline in sperm quality from 1940 to date has been well documented [2, 3] in association to increased environmental pollution and changes in the lifestyle [4]. In this regard, it is important to consider the contribution of electromagnetic fields (EMFs), since decline in sperm quality has been correlated to the level of industrialization and urbanization, rather than to a specific job [4, 5]. Further, it should be considered that Wi-Fi-equipped mobile phones and personal computers are typically positioned close to the reproductive organs. Being reproductive functions highly sensitive to microenvironment perturbations, effects on fertility potential can be also considered as an early sign of environmental hazard. In addition, a recent study on pregnant women with different habits in mobile phone use revealed that utilization for more than 1 h per day may affect biochemical parameters of cord blood, evaluated after delivery [6, 7].

In this review, we provide evidence that EMFs affect cell physiology mainly by altering redox *milieu* by influencing ROS production, antioxidant response, and mitochondrial functionality. Considering the importance of redox-related processes in the acquisition and maintenance of the biological competence of oocyte and sperm [8, 9], the purpose of the present contribution was to review the existing literature regarding the effects of EMFs on reproductive systems. Given the specifics of mitochondrial energy production during reproduction and the role of these organelles as the main source of reactive oxygen species (ROS), we focused on the hypothesis of a mitochondrial basis of EMF-induced reproductive toxicity.

## 2. Physical Basics of EMF

Electromagnetic waves are characterized by simultaneous oscillations of both electric field (EF) and magnetic field (MF). Electromagnetic waves may be characterized by parameters such as frequency (*f*), wavelength (*λ*), and electric/magnetic field strength [10]. The “frequency” describes the number of complete cycles per second, and the Hertz (Hz) is the common unit of measurement, while the term “wavelength” describes the distance over which the wave's shape repeats. A negative correlation between wavelength and frequency exists: the higher the frequency, the shorter the wavelength. When an electrical charge, positive or negative, exists, an EF is generated, whereas every moving electric charge generates a MF. Unlike EF, MFs are not easily shielded by walls and buildings [11].

Humans are exposed everyday to several natural and human-made sources of EMFs. In particular, many human activities have increased the biosphere pollution due to electromagnetic fields (EMFs) that include extremely low-frequency electromagnetic fields (ELF-EMFs), radio waves, and microwaves (MWs) [11, 12].

Natural sources generate EMFs with intensities several orders of magnitude smaller than those produced by human activity [10]. In particular, the production, distribution and use of electricity involve the generation of electric and magnetic fields, and such fields are ubiquitous in the modern societies. In addition, the impressive evolution and diffusion of mobile communication systems and wireless network technologies are dramatically increasing the exposure of humans to radiofrequency radiation and microwaves [10].

## 3. EMF and Health Effects

Among the electromagnetic fields of major interest for the potential impact on health are the ELF-EMFs and the radiofrequencies (RF). Unlike radiofrequencies, at extremely low frequencies, the electric field and the magnetic field propagate independently, and the shielding of the electric field is much simpler than that of the magnetic field, so many studies concerning electromagnetic pollution have focused their attention on EMF-induced bioeffects [13, 14]. Furthermore, it is worth mentioning that the level of penetration of the magnetic field in biological tissues is higher at lower frequencies [11, 14].

EMFs with very low frequencies (VLFs) or extremely low frequencies (ELFs) have been the object of considerable research efforts aimed at addressing the question as to whether they can cause acute or chronic diseases. Several studies suggest that ELF-EMFs do not act as initiators in the process of carcinogenesis, because the energy of the radiation is not strong enough to break chemical bonds in biological molecules. However, it has been speculated that the ELF-EMFs could have a role as co-carcinogens in the promotion and progression of cancer [15-17]. Unfortunately, conclusive information about the link between EMF exposure and cancer is difficult to obtain, given the countless reports and papers published over the decades, with different biological models, diverse physical exposure settings, and controversial findings [17]. Furthermore, potential confounding factors in the epidemiological analysis still remain an unresolved issue [18].

Beyond cancer, evidence of a correlation between exposure to different forms of EMFs and increased risks of other diseases, such as neurodegeneration and autism, is growing [19-23].

The radiofrequency (RF) portion of the electromagnetic spectrum includes MWs, along with the frequencies mostly used for wireless communication (radio and television broadcast, mobile phones, radar, Bluetooth, Wi-Fi, etc.) [21]. The most common frequencies of mobile phones used in the United States ranges from 900 to 1900 MHz. In this regard, the new network standard for next-generation wireless technology (i.e., 5 G) was finalized in June 2018, thus representing a very new milestone for the upcoming mobile infrastructure, operating at frequencies between 30 and 300 GHz [24].

For both RF and MW exposure, the characteristic parameter that identifies the interaction with biosystems is the specific absorption rate (SAR), which is expressed as the power (W) deposited by an electromagnetic radiation in a unitary mass (g) of the biological target, in a fixed time period (s). SAR is expressed as W/kg. Research has shown a negative impact of RF radiations on neurodevelopment, blood-brain barrier integrity, neurite outgrowth, neurotransmitter release, cognitive impairment, and behaviour [25–28]. Moreover, RF radiations exhibit other cellular effects such as alterations of intracellular molecular pathways, impairment of extracellular signal-regulated kinase pathway, apoptosis, and deregulation of the cell cycle [29, 30].

## 4. Biological Aspects of EMF Action: Reactive Oxygen Species, Oxidative Stress, and Mitochondria

Among the potentially toxic by-products formed in aerobic cells, reactive oxygen species (ROS) are certainly the most fascinating and most studied ones [31]. It is well recognized that low levels of ROS may play a critical role as second messengers, triggering signalling cascades, which lead to cell proliferation, apoptosis, and other key cellular processes. However, uncontrolled ROS production or impaired ROS scavenging can lead to oxidative damage to membrane phospholipids, nucleic acids, and proteins, which can disrupt normal cellular processes and trigger severe cell dysfunctions [32, 33].

In order to protect cells and organs against ROS, living organisms have evolved a highly sophisticated antioxidant protection system, which use both enzymatic and nonenzymatic strategies [34]. Antioxidant enzymes include catalase (CAT), thioredoxin reductase, superoxide dismutase (SOD), and glutathione peroxidases (GPx) [35], whereas nonenzymatic antioxidants include low-molecular-weight ascorbic acid, tocopherols, tocotrienols, melatonin, carotenoids, natural flavonoids, and thiol-containing antioxidants, among which tripeptide glutathione (GSH) plays a crucial role, being an essential cofactor for several antioxidant and detoxification enzymes [36]. Given the tight correlation between the production of ROS and mitochondrial function, a growing interest is emerging on the role of such organelles in the responses evoked by EMF insult in biosystems.

Several studies showed that the exposure of different cell types to a 50 Hz ELF-EMF induced an increase in intracellular ROS levels [37–40] that was able to trigger a temporary mitochondrial permeability transition [41, 42]. By contrast, Luukkonen and coworkers [43] showed that an increase in mitochondrial activity induced by ELF-EMFs could be the cause of the raise in ROS and lipid peroxidation levels in the SH-SY5Y cell line. Moreover, neuroblastoma cells exposed to ELF-EMFs for 5 days exhibited decreased mitochondrial respiration, whereas these cells treated for 15 days showed enhanced aerobic mitochondrial function and biogenesis [44–46]. Recently, it has been proposed that ELF-EMFs may reduce proton leaks or affect ATP-demanding events, by inducing a compensatory transient increase in mitochondrial respiratory activity [47]. Nevertheless, other studies did not observe these effects on mitochondrial potential after ELF-EMFs, probably due to differences linked to the field intensity and experimental model employed [48]. Similarly, numerous authors did not find the increase in ROS levels described above [44, 49–51]. In particular, the exposure to low-intensity ELF-EMFs was able to diminish ROS levels in dermal fibroblasts, human keratinocytes [40, 52], and neutrophils [53].

Several reports evaluated also the influence of the ELF magnetic field on the enzymatic and nonenzymatic antioxidant defence systems [17, 54–59]. Manikonda and colleagues [60] found that rats exposed to ELF-MF for 90 days continuously showed oxidative stress with an increase in lipid peroxidation end products and altered glutathione (GSH/GSSG) levels in a dose-dependent manner in various regions of the brain. On the contrary, other works suggested that ELF-EMF could lower SOD, CAT, and GPx activities in rodent brain [61–63]. A recent work of our group demonstrated that human neuroblastoma cells responded to a long-term 50 Hz, 1 mT ELF-EMF by increasing CAT- and GPx-dependent scavenging efficiency, as well as by improving the availability of reduced glutathione, together with the upregulation of three of the major redox-responsive controllers of cellular response towards oxidative stress (i.e., sirtuins 1/3, and NRF2), thus resulting in a reduced vulnerability against ROS-based treatments [59].

Besides ELF-EMFs, RF-EMFs have been under the researchers' attention for decades, as a potential threat to human health, with a particular focus on their capacity to promote ROS production and oxidative stress [64–72]. Apart from few discordant studies [73, 74], it is widely recognized that RF exposure induced a significant increase in intracellular ROS levels, as well as lipid peroxidative products, along with the downregulation of transcriptional and protein levels of antioxidant genes, such as SOD1, SOD2, CAT, and GPx1 in different cell lines [75–78]. These effects were associated with a time-dependent decline in mitochondrial potential, increased mtDNA damage, and reduction in mtDNA copy number and mtRNA transcript level [79–82]. Mitochondrial alterations have been ascribed to ROS-related mutation at the origin of replication and transcription in the mtDNA or to alterations of the mtDNA nucleoid structure [79, 82]. Moreover, the observation of reduced intracellular ATP content supports the hypothesis that less functional mitochondria needed time to restore their original capacity to synthesize ATP to the control levels [82].

Consistently, pretreatment with antioxidant compounds, such as melatonin, was able to detoxify ROS, prevented mtDNA damage, and stimulated antioxidative enzymes in different pathophysiological states, especially in the brain [79, 83, 84].

Recently, it has been proposed that mitochondria could represent one of the targets of noninvasive RF treatment for pancreatic tumors [85]. Indeed, Curley and colleagues [85] demonstrated that RF induced changes in the shape of mitochondrial cristae of human pancreatic cancer cells, by altering the polarization of mitochondrial membrane, impairing the oxidative respiration, and increasing ROS production, thus supporting the hypothesis of RF-EMF-induced stress on mitochondria.

Taken together, most of these results show that EMFs are able to induce alterations in ROS production, antioxidant response, and mitochondrial imbalance, although these effects seem to be strictly related to the experimental set-up, including the EMF shape/intensity/frequency/exposure time and different cell lines and tissues.

## 5. EMF Effects in Male Reproductive System and Fertility

The male reproductive system is under regulation of complex processes based on the cross-talk among the hypothalamus, pituitary gland, and testis, which is the site of spermatogenesis. Endocrine stimulation of this process is operated by follicle-stimulating hormone (FSH) and luteinizing hormone (LH), which acts by means of testosterone produced by Leydig cells in the testis. Sertoli cells and peritubular cells play a main role in these complex signalling processes where, in addition to hormonal signals, a plethora of growth factors and cytokines act to control the mitotic and meiotic phases of spermatogenesis. At maturity, A1 spermatogonia undergo an asymmetric division resulting in the formation of another A1 spermatogonium and a type 2 spermatogonium, which divides to produce A3 spermatogonia, followed by A4 spermatogonia and then intermediate spermatogonium. This cell divides to form type B spermatogonia that generate the primary spermatocytes, which undergo the first meiotic division to produce secondary spermatocytes. These cells complete the second meiosis division to form the aploid cells also known as spermatids. During the divisions described above, the cells migrate from the basal membrane of seminiferous tubules towards the lumen. Spermatids undergo a complex cytodifferentiation, during which active gene transcription ceases, the chromatin is remodelled, and the assembly of sperm flagellum occurs to form spermatozoa that will ultimately be released from the seminiferous epithelium via a process known as spermiation [86, 87].

Throughout spermatogenesis, mitochondria undergo peculiar morphological changes in concomitance with increased metabolic requirements [88]. During spermiogenesis, the number of these organelles is severely reduced, and the remaining mitochondria form the mitochondrial sheath around the axonemal complex and the outer dense fibers of the middle piece [89]. Although sperm mitochondria are destroyed within the fertilized oocyte, their functionality is crucial for the process of fertilization and embryo development. Loss of sperm functions has been correlated with genomic, transcriptomic, and proteomic mitochondrial alterations [90]. Particularly sensitive to mitochondrial dysfunctions are the events, which follow the epididymal storage including passage in the female reproductive tract and sperm capacitation. Searching for the roles of mitochondria in sperm functions, it emerges that mitochondria-derived ATP is not essential for sperm motility that rather relies on glycolysis-derived ATP [91]. A relevant role of sperm mitochondria is associated with the production of ROS, which at controlled levels can properly regulate sperm function [92]. Sperm are particularly susceptible to oxidative stress due to limited cytoplasmic antioxidant capacity and abundance of sensitive ROS targets including DNA, thiol-rich proteins, and polyunsaturated fatty acids (PUFAs) with a main role in supporting sperm motility and fusion during fertilization [93]. Thus, increased levels of mitochondrial ROS are hypothesized to activate a feedback loop leading to increased lipid peroxides and DNA oxidation gradually compromising sperm functions and initiate apoptosis [94].

### 5.1. Impact of ELF-EMFs

Many experimental studies have been carried out in order to evaluate the risk of ELF-EMFs on male reproductive function and sperm quality in mammalian species including humans, but data obtained are often contradictory. Nevertheless, apart from the individual endpoints considered in different studies, there is general consensus regarding the negative impact of ELF-EMFs on the fertility potential of exposed males. Contradictions may be due to the different experimental settings or animal models or even explained by different impacts of ELF-EMFs in animals of different sizes [95]. It was observed that male mice exposed to 50 Hz ELF-EMF for 90 days were able to impregnate unexposed females with the same potential of sham control [96, 97]. By contrast, in the rat model, a reduced reproductive outcome was observed when males exposed to 50 Hz ELF-EMF for 90 days were mated with unexposed females soon after removal of the field, or 45 days later [98]. Indeed, although soon after the ELF-EMF exposure only half of the males were able to impregnate the female, the fertility potential improved after 45 days and was recovered after 90 days from ELF-EMF exposure. Moreover, although similar numbers of viable foetuses were observed in all experimental groups in comparison to control, the number of foetus resorptions increased regardless of postirradiation time. Nevertheless, the authors suggested that resorptions soon after ELF-EMF exposure could be ascribed to poor embryo competence, while resorptions after 90 days could be a bias linked to reduced quality of semen due to male aging. Furthermore, prolonged ELF-EMF exposure induced reduction of sperm count and serum testosterone level, which is probably the cause for reduced functionality of seminal vesicles and preputial gland observed after ELF-EMF [99, 100].

According to some studies, the intensity of exposure may have an important role in the quantity and quality of the effects in the male reproductive system. Indeed, the exposure to 5 *μ*T, 83.3 *μ*T, or 500 *μ*T doses at a frequency of 60 Hz during foetal life and early life was reported to have no impact on anogenital distance, preputial separation, testis weight, and testicular cell populations of newborn rat males [101]. By contrast, a 1 mT, 60 Hz dose during the same period caused a delay in testis development, characterized by reduced volume of seminiferous tubules and seminiferous epithelium. This effect was also observed in mice born from dams exposed to 50 Hz at 500 mG for 1 week [102] and may be ascribed to a reduced functionality of Sertoli cells, which are deputed to support epithelium growth and blood-testis barrier [103]. Furthermore, a reduction in number of Leydig cells with no effects on testosterone levels has been reported [103]. By prolonging the exposure to 90 days of age, ultrastructural studies demonstrated that testicular degeneration was more marked in comparison to rats exposed until 21 days of age, suggesting the ability to repair damage and activate spermatogenesis after a postexposure recovery [104]. In particular, ELF-EMF testes were characterized by germ cells with extended abnormal morphology such as vacuolization and signs of apoptosis like fragmentation, condensed nucleus, and apoptotic bodies [104, 105]. Cytoplasmic vacuolization was also observed in Sertoli cells, and some Leydig cells showed alterations in chromatin condensation that may be interpreted as an apoptotic phenotype [104, 106]. Ultrastructural studies reporting apoptotic modifications of cells of seminiferous tubules are in accordance with DNA apoptotic damage observed in testes of mice exposed to 60 Hz [107, 108]. Although further investigation is required to clarify the mechanism by which ELF-EMF exerts its deleterious effects, some authors have suggested that increased apoptosis may be a mechanism to prevent the maturation of abnormal germinal cells that may lead to the formation of defective sperm [104, 105, 107–109]. An important feature shared by all cell populations of seminiferous tubules was the presence of abnormal mitochondria, which appeared more electron-dense and lost their cristae. Mitochondria of elongating spermatids did not show the normal well-organized distribution after ELF-EMF exposure [104]. Accordingly, alterations in mitochondria functionality induced by ELF-EMFs have been suggested as the main cause of reduced sperm motility and vitality observed in rodent and human spermatozoa [97, 110, 111].

In contrast to the results reported above, some studies discovered beneficial effects of ELF-EMF on male reproductive potential. Hori and coworkers [112] observed that males of a mouse strain with a low reproductive performance increased their copulation rate when exposed to ELF-EMF for 11 days prior to caging with superovulated females. Since this occurred without alterations of plasma levels of testosterone, LH, and nitric oxide, the authors suggested that ELF-EMF exposure may be beneficial for the treatment of male sexual dysfunctions [112]. Finally, in a revision of the literature, Darbandi et al. [113] hypothesized that exposures of a few hours to 50 Hz ELF-EMF with a square waveform may be associated to beneficial effects of sperm morphology and motility, whereas longer periods of exposure or ELF-EMFs with different characteristics may negatively influence sperm quality or have no effects.

### 5.2. Impact of RF-EMFs

In 2009, a large study that followed the growth, development, and fertility potential of mice living under constant exposure to three doses of RFs (in particular, Universal Mobile Telecommunications System (UMTS)) over four generations of mice reported no effects on the male reproductive system in terms of appearance of reproductive organs, fertility potential, and offspring development [114]. The lack of embryo toxicity and teratogenic effects was also confirmed by studies conducted in rats exposed to 2.14 GHz or Wi-Fi signal during gestation and lactation and followed over two generations [115–117].

By contrast, other studies reported a reduction in litter size obtained from males exposed to RFs and mated with unexposed females in comparison to control rats [118, 119]. These effects were associated with decreased weight of testes, disorganized seminiferous tubules, reduced testosterone levels, and production of apoptotic damaged sperm [119–121]. In particular, a marked reduction in germline cells, hypospermatogenesis, maturation arrest, and increased sperm with morphology alterations were observed after 7 days of RF exposure [122]; a decline in testis weight with increased DNA damage, marked reduction of seminiferous tubule diameter, and drop in sperm production and quality were reported under prolonged exposures [123–128]. Ultrastructural analysis revealed that sperm produced after RF were characterized by distortion in the head and alterations in mitochondrial distribution in the mid-piece [119, 127]. Since a proper mitochondrial distribution has a key role in sperm motility, these alterations may account for the reduced sperm motility reported after RF [124, 129]. Sperm obtained from mice exposed to RF for 30 days had a reduced ability to fertilize oocytes *in vitro* and lead to reduced quantity and quality of embryos at blastocyst stage [130].

A plethora of studies focused on the impact of RFs on human spermatozoa [67, 131–135]. The increased use of mobile phones in the 2000s has led researchers to investigate the possible relationship between daily mobile phone use and semen parameters [131, 132]. These results were concordant in highlighting a decrease in the quality of sperm obtained from men using mobile phones, with more pronounced effects in heavy users [131, 132]. Afterwards, in most studies, human sperm donated from normozoospermic volunteers were exposed to RF emitted by mobile phone, laptop Wi-Fi, or other sources and then evaluated after a few hours for the presence of effects on sperm parameters [67, 133–135]. In some cases, samples were employed after selection of highly motile sperm [134, 135]. As a result of the similar experimental settings, all the studies reported above agreed on the detrimental effects of RFs on sperm vitality, motility, and morphology.

### 5.3. EMF-Associated Oxidative Stress and Male Fertility: Role of Mitochondria

Although the mechanisms by which EMFs can interact with biological systems are not yet understood, increasing evidence leads us to assume that EMFs can interfere with the cellular oxidative/antioxidant balance, both *in vitro* and *in vivo* [56, 136–138]. Oxidative stress is one of the most recognized causes of male infertility, and this is exacerbated by the fact that spermatozoa are especially vulnerable to oxidative stress [139]. There is a growing body of evidence suggesting that EMF exposure during spermatogenesis induces a redox imbalance due to both an increase of ROS production and a decrease in ROS scavenging activity (Table 1). A plethora of studies demonstrated that prolonged RF exposure caused an increase in ROS production and an imbalance in total antioxidant capacity in terms of reduction of GPx, CAT, and SOD which leads to increased lipid peroxidation in rat sperm and testes [140–143]. The resulting imbalance in the redox status altered the sperm cycle progression and activated the apoptotic program through the reduction of bcl-2 expression and the raise of bax, cytochrome c, and caspase 3 protein and gene expression [140, 141, 144]. Interestingly, diet supplementation of vitamins C and E reverted the reduction in GSH level and GPx activity and the increase in lipid peroxidation observed in testes of rats exposed for 14 days to a RF (900, 1800, and 1900 MHz, SAR 0.9 W/kg) [124, 145]. Similarly, diet supplementation with melatonin and *Moringa oleifera* leaf extract prevented alterations in sperm count and motility, increased lipid peroxidation and DNA damage, and decreased intracellular GSH levels and SOD enzymatic activity recognized after 902.4 MHz RF, SAR 0.0516 W/kg [122, 146].

Exposure of *in vitro* mouse spermatogenesis pathway cell lines (GC-1 spg and GC-2 spd) to 50 or 120 Hz ELF-MF determined an increase in superoxide anion production, which was associated to activation of the intrinsic apoptotic pathway and proliferation inhibition, although after a 48-hour postexposure recovery, an increased activation of NF-*κ*B, a transcription factor involved in the maintenance of redox balance, was found [147, 148]. These effects were abolished by medium supplementation with Aloe arborescens's juice [148].

Similarly to RFs, Saadeldin and colleagues [149] reported that a 50 Hz, 100 *μ*T ELF-MF exposure for 21 days caused a reduction in SOD and CAT activity, along with a decrease in rat epididymal sperm motility and vitality. Since a partial recovery of redox imbalance was recognized after a 48-day postexposure, a transitory ELF-EMF effect was hypothesized. By contrast, several authors demonstrated that long-term exposure of rat to ELF-MF did not affect sperm count and morphology and oxidative and antioxidative parameters such as malondialdehyde (MDA) levels, total antioxidant capacity (TAC), and CAT and SOD activity although increased levels of apoptotic markers were observed [109, 150].

Spermatozoa are particularly vulnerable to oxidative stress because of their limited cytoplasmic volume restricting the availability of intracellular antioxidant defences [151]. In humans, a close correlation was observed between reduction in sperm motility and decrease in mitochondrial membrane potential [152, 153]. De Iuliis and colleagues [133] demonstrated that an overnight exposure to RF significantly increased the amounts of ROS in functional human spermatozoa and that the site of production of these free radicals was mainly the mitochondria. Indeed, they showed a strong correlation between ROS production and electron leakage from the mitochondrial electron transport chain. Furthermore, when human spermatozoa were exposed to increasing levels of RF-EMFs, a significant dose-depending mitochondrial ROS generation was observed. As a result, increased levels of 8-OH-dG, the main marker of oxidative DNA damage, were observed and associated with reduction in both sperm vitality and motility [133]. Moreover, Pandey et al. [154] demonstrated that mice exposed to 900 MHz EMF for 4 or 8 h daily for 35 days showed an increase in the number of germ cells with alterations of electron potential of mitochondrial membrane. Houston and colleagues [155] also showed that a 4 h exposure of mouse spermatogonial and spermatocyte cell lines to RF-EMF induced the generation of mitochondrial ROS, probably produced by complex III of the electron transport chain, able to increase DNA oxidative damage and fragmentation. According to the authors, the depolarization of the mitochondrial membrane could be the trigger for the ROS generation which in turn caused the DNA breaks observed in exposed mice. In a case-control study, Hagras et al. [156] found that patients with prolonged cell phone use (>4 h daily) showed a significant imbalance in sperm motility. In the same work, it was revealed that mitochondrial isoenzyme NAD^+^-dependent isocitrate dehydrogenase (IDH) activity was increased in the seminal plasma of exposed patients. It is known that this isoenzyme is localized only in the mitochondrial matrix, where it regulates the ATP generation in the TCA cycle. Thus, the same authors speculated that the NAD^+^-dependent IDH in seminal plasma could be due to a probable mitochondrial loss of this protein after EMF-related damage. As shown by Aitken et al. [157], a 900 MHz RF-EMF exposure for 12 h/day for a week at a SAR of 90 mW/kg significantly caused genotoxic effects on mitochondrial DNA in mouse epididymal spermatozoa. They chose this temporal range of exposure to collect spermatozoa from cauda epididymis or rather during epididymal transit because at this stage spermatozoa are more vulnerable to genotoxic factors.

On the other hand, other researchers reported no significant EMF effects on spermatozoa. Falzone and colleagues [158] demonstrated that a 1 h exposure to a pulsed 900 MHz GSM mobile phone radiation at an average SAR of either 2.0 or 5.7 W/kg and at constant temperature did not result in mitochondrial membrane potential changes in human spermatozoa. However, they found a decrease in all velocity parameters in RF-EMF-exposed spermatozoa at 5.7 W/kg SAR value when compared to controls. The same authors observed a correlation between an increase in intrinsic ROS generation and a decrease in sperm motility at the higher SAR level, so they hypothesize that mitochondrial membrane potential could not be correlated with ROS production in human spermatozoa.

Bernabò et al. [159] reported that a 4 h exposure to a 50 Hz, 1 mT ELF-MF significantly decreased intracellular Ca^2+^ storage together with lower mitochondrial activity and spermatozoa motility in boar. EMF exposure is able to generate free radicals, and this fact would seem to produce structural aberrations. In 2011, Tenorio and colleagues [103] described how rats exposed to a 60 Hz, 1 mT ELF-MF from gestation to adult period showed spermatids with mitochondria near the acrosome and not around the mitochondrial annulus. Moreover, the same work reported the presence of extremely electron-dense mitochondria with loss of cristae in rats exposed to spermatids. An electron microscopy study performed by Khaki et al. [160] showed that a pre- and postnatal chronic exposure to 50 Hz, 8 mT EMF induced cell damage including mitochondrial dilatation in stromal cells of rat prostate glands. Conversely, Iorio et al. [161] gave evidence for a stimulatory effect on human sperm motility exerted by a 50 Hz, 5 mT ELF-MF exposure in association with a greater activity of mitochondrial metabolism efficiency. On the other hand, Solek et al. [148] reported no significant effects of EMF exposure (50 Hz, 2.5 mT, for 2 h) on mitochondrial function in mouse germ cells.

As summarized in Table 1, we can conclude that both ELF- and RF-EMF exposure could be crucial factors responsible for mitochondrial ROS production in spermatozoa. Indeed, EMFs could induce perturbations to the mitochondrial proton motive force with subsequent impairment of the balance between ROS generation and ROS scavenging [162]. The resulting oxidative stress could play a pivotal role in the mitochondrial damage causing the loss in sperm motility, one of the most significant causes of fertility impairment.

**Figure 1:**
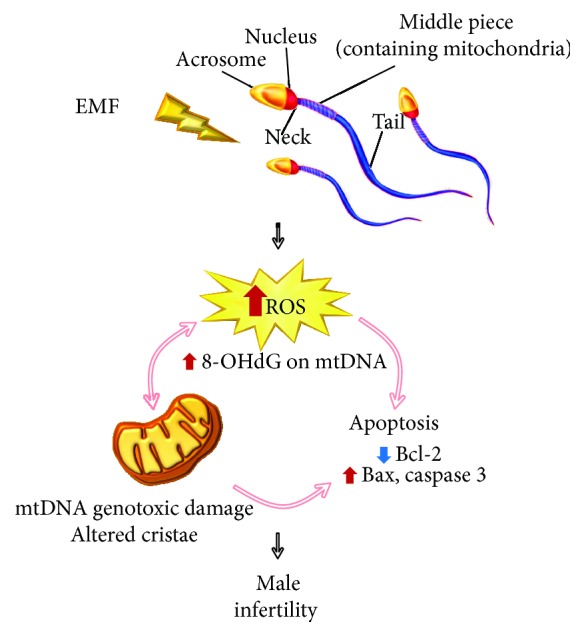
Involvement of oxidative stress and mitochondrial dysfunction after EMF exposure in the male reproductive system. EMF: electromagnetic fields; ROS: reactive oxygen species; 8-OHdG: 8-hydroxy-2′-deoxyguanosine; Bcl-2: apoptosis regulator Bcl-2; Bax: Bcl-2-associated X protein.

The involvement of both mitochondrial metabolism and oxidative balance can suggest that ELF-EMFs could act as an environmental trigger for the intrinsic apoptosis pathway during spermatogenesis, although more studies are needed to understand the molecular mechanisms involved (Figure 1).

## 6. EMF Effects on Female Reproductive System and Fertility

The female reproductive system is a fine-tuned system that relies upon precise coordination of a complex series of interactive events involving hypothalamus, pituitary gland, ovaries, and uterus. Under hypothalamic GnRH (gonadotropin-releasing hormone) pulses, the pituitary gland activates the release of gonadotropins, FSH and LH. FSH activity is critical for recruitment and maturation of ovarian follicles, expression of the LH receptor, and differentiation of follicle cells in outer theca cells and inner granulosa cells. Theca cells provide androgens to granulosa cells that operate androgen conversion to estradiol, whose increase contributes to the onset of LH surge and the inhibition of FSH secretion. The peak of LH triggers oocyte resumption of meiosis, progression to the second meiotic metaphase, and ovulation of the oocyte-cumulus complex. Then, estradiol levels decline and luteinization of the follicle increases production of progesterone, reducing GnRH/LH pulse frequency and preparing the endometrium for embryo implantation [163]. The functional unit of the ovary is the ovarian follicle formed by the oocyte and surrounding follicle cells. Once recruited in the growing phase, primordial follicles develop into primary, secondary, and eventually antral follicles reaching the preovulatory stage under gonadotropin stimulation. The antral follicle is characterized by the presence of an antrum filled with follicular fluid bathing an oocyte surrounded by cumulus cells [163]. The pool of primordial follicles, also known as ovarian reserve, is nonrenewable and provides fertilizable oocytes from the onset of puberty to menopause when primordial follicles are exhausted [164]. An accelerated depletion of ovarian reserve can be caused by exposure to environmental insults during reproductive lifespan with huge effects on female fertility [165, 166].

### 6.1. Impact of ELF-EMFs

When focusing on ELF-EMF effects on female fertility potential, data present in literature are rather conflictual. In rats, exposure to a 50 Hz sinusoidal magnetic field of approximately 25 *μ*T for 90 days before mating significantly reduced their fertility in terms of reduced number of implantations and living foetuses per litter [98]. By contrast, when female mice were exposed to the same electromagnetic field (50 Hz, 25 *μ*T for 90 days), the number of pregnant females, implantations per litter, and viable foetuses was similar to control [96]. Mice presented an increase in ovarian weight in association with similar body and uterine weight [96]. In rats, two studies demonstrated that cumulative ovarian and uterus weight increased after 1 month of exposure, decreased to normal levels after two months, and was lower than control after 3 months of exposure [167, 168]. Apart from differences linked to the use of two rodent species, these contradictory results may be explained by the fact that induced eddy currents generated by the same alternating field intensity may depend on the size of the animal models [95].

Exposure to the ELF-EMFs from womb to adult life reduced weight at birth, delayed weight gain, and puberty onset in females in comparison to exposure started at birth or sham conditions in mice and rats [102, 169]. When focusing on the effects of the ELF-EMFs on folliculogenesis and ovarian reserve of pups born from dams exposed to a 50 Hz ELF-EMF and grown to adulthood in normal conditions, histological analysis showed that newborn pups presented a reduced number of primordial follicles cysts, most of them showing abnormal follicle or pregranulosa (stromal) cells. Ultrastructural studies revealed that oocytes from ELF-EMF pups presented irregular nucleus with condensed heterochromatin and numerous vacuoles in the cytoplasm [170, 171]. When female mice exposed to ELF-EMFs *in utero* grew to adulthood, their ovarian reserve was reduced in association with an increase in atretic follicles. These follicles were characterized by abnormal oocytes with zona pellucida damage and granulosa cells with absent or condensed mitochondrial cristae and separated from surrounding stromal cells and vacuolization of theca interna [170]. Similar features were observed in ovaries from rats exposed to ELF-EMFs during foetal and adult life [169, 172, 173], suggesting that exposure to ELF-EMFs during embryonic development induces morphological changes that affect irreversibly folliculogenesis, inducing loss of oocytes, degeneration of granulosa cells, and decreased ovarian reserve, leading to a condition of subfertility.

*In vitro* studies on preantral ovarian follicles demonstrated that ELF-EMF exposure affected the ability of follicles to develop antral cavities, and this detrimental effect was associated with the production of oocytes with a reduced ability to undergo meiosis resumption. Nevertheless, it seems that ELF-EMFs did not affect mechanisms controlling meiotic maturation *per se* since preovulatory cumulus-enclosed oocytes obtained from primed mice and exposed to ELF-EMFs reached the MII stage during *in vitro* culture at a similar extent as control [174]. Moreover, ovulated mature oocytes exposed to 4 kHz pulsed ELF-EMFs for 5 h after IVF showed fertilization rates similar to controls [175]. Taken together, this evidence supports the conclusions that ELF-EMFs affect folliculogenesis by targeting the ovarian and follicle microenviroment. As consequence, oocytes lack proper support during growth and differentiation [170, 172, 174]. Moreover, oocyte susceptibility to ELF-EMFs seems to be reduced once reaching the preovulatory stage.

**Figure 2:**
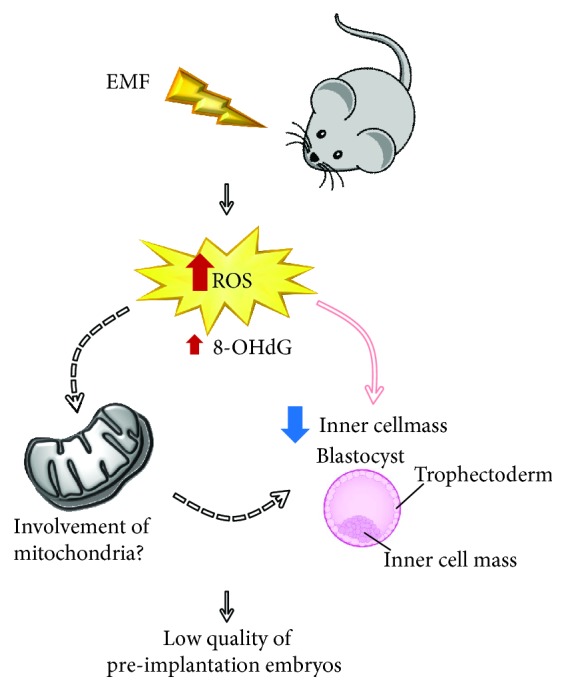
Involvement of oxidative stress and possible role of mitochondria-related pathways in embryos produced from females exposed to EMF. EMF: electromagnetic fields; ROS: reactive oxygen species; 8-OHdG: 8-hydroxy-2′-deoxyguanosine.

By focusing on the effects of ELF-EMFs during mating and early stages of pregnancy, similar numbers of pregnant mice were observed [176, 177]. Nevertheless, Borhani and colleagues [176] reported the harvest of fewer blastocysts in the ELF-EMF group in comparison to the control. Moreover, although presenting similar numbers of blastomers, blastocysts from ELF-EMF-exposed mice presented an increase in DNA fragmentation (Figure 2). By contrast, Bayat and coworkers [177] observed no differences in the number of collected blastocysts, but described a reduction in cells composing the blastocyst with a prominent reduction in inner cell mass component. Collectively, these works highlighted that ELF-EMF exposure during preimplantation embryo development affects reproductive outcome by decreasing the quality of blastocysts produced [176, 177].

Alterations of the reproductive performance caused by ELF-EMFs may also be ascribed to effects on the hypothalamic-pituitary-gonadal (HPG) axis [178]. In rats, ELF-EMFs induced a transient increase in progesterone level and a transient decrease in estradiol, FSH, and LH. Normal levels of these hormones were recovered after a longer exposure time or the removal of the field [167–169]. A reduction in IGF1 in females exposed from foetal life may be responsible for the reduction in growth and delay in sexual maturity observed in these rats [169].

### 6.2. Impact of RF-EMFs

The impact of RF-EMFs on female reproduction and fertility has been so far poorly investigated. The main reason behind this lack of interest is that most of the studies in the field highlighted no differences or hotspots. In 2009, Sommer and colleagues [114] investigated the impact of three doses of RFs (UMTS) over four generations of mice without observing any anomalies in reproductive organs, fertility potential, and offspring development, apart from a slight reduction in food intake in the generation of F0- and F1-exposed groups, although without a clear dose-response relationship. In accordance, female rats exposed to low or high RF doses during pregnancy and lactation presented no abnormalities in terms of growth, gestational condition, and organ weights. The F1 generated from these females showed survival rates, development, growth, physical and functional development, hormonal status, memory function, and reproductive ability similar to sham control, and no teratogenic effects were observed in the generation of F2 [115–117]. Neither the exposure to the very high level of 4 W/kg was demonstrated to have deleterious effects on newborn developmental potential [117, 179]. By contrast, Sangun et al. [180] reported a reduction in postnatal growth and delay in puberty onset in female Wistar rats exposed to 900 MHz during foetal life. Interestingly, low postnatal growth was associated with increased food consumption and similar levels of IGF1, suggesting an effect of RFs on rat metabolism. Since normal pubertal development is strictly linked to the interplay between central and peripheral signals in order to have the pulsatile release of LH and FSH, the authors investigated the hormonal profile of these rats exposed during foetal life. Nevertheless, in addition to delayed puberty onset, increased serum LH levels were observed, a condition associated to accelerated loss of primordial follicles in the adult [181], whereas no differences in ovarian follicle count were revealed [180].

Several studies demonstrated detrimental effects of RF on ovarian follicle population. Gul and coworkers [182] placed a mobile phone under the cages of female rats during the entire pregnancy for 11 h and 45 min in standby and 15 min in speech mode. This exposure affected the volume and the primordial follicle content of newborn ovaries sacrificed on the 21^st^ day after delivery. However, a great limitation of this study was the lack of measurement of the amount of MWs emitted by the cell phone. To avoid bias linked to animal movements inside the cage, Bakacak and colleagues [183] used an experimental device to apply RF-EMF of 900 MHz directly on the abdomen region of female rats for 15 min/d for 15 days. In these conditions, the observation of a significant reduction of ovarian reserve in terms of reduction of primordial ovarian follicles in RF-EMF rats was confirmed.

Females born from dams exposed to 900 MHz RF-EMF during pregnancy presented severe ovarian damage, with a reduction of primordial and tertiary follicle, increase of atretic follicle, pronounced vasocongestion, and stromal fibrosis. Moreover, most of primary follicles had lost their oocytes and granulosa cells presented extended degeneration, vacuolization, and loss of connection with cumulus cells [184], as observed after exposure to ELF-EMFs. When looking for mechanisms behind ovarian damage and follicle loss, the authors observed an increase in apoptosis in ovaries from exposed rats, suggesting that the activation of the programmed cell death machinery may be responsible for impairment of the follicle development process and decrease of ovarian follicle reserve [184].

Finally, exposure of mouse oocytes and sperm to RF for 60 min prior to *in vitro* fertilization techniques had no effects on fertilization rates, blastocyst formation, and chromosomal aberration [185].

### 6.3. EMF-Associated Oxidative Stress and Female Fertility: Role of Mitochondria

The impact of oxidative damage on RF effects in the female reproductive system was investigated in ovaries from female rats exposed to 900 MHz during foetal or postnatal life. Total antioxidant status (TAS), total oxidant status (TOS), and oxidative stress index (OSI) were increased in RF ovaries regardless of time of exposure. Although the exposure conditions did not alter ovarian follicle population, increase in TAS, TOS, and OSI values highlighted the oxidative nature of the stressing condition experienced by the ovary under RF exposure [180]. This is in accordance with studies in nonmammalian models. An interesting contribution to the knowledge on oxidative stress impact on ovarian physiology came from a study on *Drosophila melanogaster* [186]. Genome-wide microarray analysis performed on early- and middle-stage follicles under RF exposure revealed the activation of more than 150 genes and the downregulation of only 15 genes involved in various cellular functions. Since a twofold increase in ovarian ROS content and activation of follicle cell death were observed, authors suggested that genome-wide and nontargeted transcriptional perturbation of gene-expression profiling, probably induced by RF-dependent ROS increase, could compel sensitive ovarian-cell subpopulations and lead to sporadic follicle apoptotis [186]. Similarly, exposure of quail embryos to 900 MHz determined overproduction of superoxide and nitrogen oxide, in association with increased level of lipid peroxidation and depression of key antioxidant enzymes activity, resulting in increased level of oxidative damage of DNA in the embryo [187]. Since authors used electron paramagnetic resonance (EPR) spin-trapping technique for the specific detection of superoxide in mitochondria, this work represents a strong evidence of mitochondrial origin of superoxide overproduction under RF exposure in embryonic cells. Nevertheless, the site of interaction of RF with mitochondria structures/electron transport chain and the mechanisms by which RF-EMR exposure induces free radical overproduction remain unclear.

Despite the well-known connection between ELF-EMFs and oxidative stress, only two studies investigated the oxidative status of females exposed to ELF-EMFs [168, 173]. Prolonged exposure to 50 Hz induced increased MDA levels in rat uterus and ovaries [173]. By contrast, Aydin and colleagues [168] observed a significant reduction in CAT serum activity, but no significant differences were found in GSH and MDA serum levels.

Finally, it is important to remark that animal models have different sensitivities to physical and chemical exposure; therefore, RF effects observed in all biological models here reported are not directly applicable to the situation in humans and should be interpreted with caution.

## 7. Conclusions and Final Remarks

Based on the current literature, the analysis of ELF-EMF and RF impact on the maintenance of male and female fertility potential reports contradictory results. The main reason for these discrepancies may be the lack of uniformity in the experimental design, including the use of different models and the extremely variable exposure sources and protocols. Moreover, since ROS levels can be influenced by temperature, a possible criticism to many of these works is the lack of control of this parameter during EMF exposure [188]. On the other hand, growing evidence suggests that the damage induced by EMFs to reproductive cells and organs is caused by deregulation of redox homeostasis [140, 144–146, 149, 168, 180, 186, 187].

Based on *in vitro* studies, there is a general consensus on the effects of EMFs from mobile phones, laptops, and other electric devices on human sperm quality with possible negative influence on fertility [67, 113, 131–135, 189–192].

The role of mitochondria as one of the main targets of ELF- and RF-EMF exposure has emerged, especially in the male reproductive system. Indeed, these radiations seem to directly target the electron transport chain thus establishing mitochondrial dysfunctions and ROS overproduction, self-reinforced in a vicious cycle [103, 133, 154, 156, 159, 187].

According to recent data reported here [122, 146, 148], the strict link between EMF-related damage of reproductive systems and oxidative stress is reinforced by the observations of protective effects of antioxidant supplementations that require to be confirmed in humans. This approach, although still unexplored in the female reproductive system, could represent an important preventive strategy.

A further criticism emerging from the literature is the difficulty to understand whether EMF-induced fertility abnormalities are caused by direct gonadal damage or by disruption of the hypothalamic-pituitary-gonadal axis. Indeed, most studies rely on total body exposure of small animals within cages. In this regard, the application of EMF-emitting devices to abdominal regions [183] or the use of large animal models may help to elucidate the mechanisms underlying ELF-EMF and RF biological effects, as suggested by Bernabò and colleagues [193]. This kind of approach would aid to clarify the impact of ELF-EMF and RF on human health, also considering the entry in our lives of the new 5G standard that may increase environmental EMF pollution.

Finally, an interesting aspect of EMF-related biomedical research that has been poorly investigated regards what happens to reproductive cells and organs when the exposure to EMFs occurs in concomitance with other environmental pollutants. This issue would be of great interest since our everyday life is characterized by the continuous presence of some environmental agents (e.g., EMFs) and the sporadic or frequent occurrence of exposure to other chemicals and physical toxicants. In support to these concerns, Tenorio et al. [106] observed that the exposure to ELF-EMF after testis heat shock irreversibly damages the spermatogenic process. Thus, EMFs may represent a risk factor for fertility in males suffering from reversible testicular damage.

In conclusion, future and more standardized studies are needed in order to understand the molecular mechanisms underlying EMF challenge to reproductive systems and establish preventive strategies.

## Figures and Tables

**(a):** 

Reference	Radiation	SAR	Exposure time	Model	Temperature	Effect
[157]	RF 900 MHz	0.090 W/kg	Long-term exposure	Mice	—	Mitochondrial DNA damage
[158]	RF 900 MHz	2–5.7 W/kg	Short-term exposure	Human spermatozoa	Constant	No effect on mitochondria
[67]	RF 850 MHz	1.46 W/kg	Short-term exposure	Human semen	Room temperature	Loss in sperm motility and viability Increased ROS production
[133]	RF 1.8 GHz	0.4–27.5 W/kg	Short-term exposure	Human spermatozoa	Constant	Mitochondrial ROS generation Alterations of electron potential of mitochondrial membrane, Oxidative DNA damage
[124]	RF 900–1800 MHz	—	Long-term exposure	Rat	Constant	Lower sperm motility Decreased GSH levels Oxidative damage
[140]	RF 900 MHz	0.9 W/kg	Long-term exposure	Rat	—	Decreased antioxidant enzyme activity Increased ROS production Lipid peroxidation Increased apoptosis
[144]	MW 10 GHz	0.014 W/kg	Long-term exposure	Rat	—	Spermatozoa cell cycle arrest ROS production
[145]	RF 900–1800–1900 MHz	0.9 W/kg	Long-term exposure	Rat	—	Decreased antioxidant defences Lipid peroxidation
[122]	RF 900 MHz	—	Long-term exposure	Rat	—	Decreased sperm count Decreased antioxidant defences Lipid peroxidation
[141]	RF 900 MHz	0.66 W/kg	Long-term exposure	Rat	—	Decreased sperm viability Increased ROS production Increased apoptosis
[156]	RF 1800–1900 MHz	—	Long-term exposure	Human	—	Mitochondrial damage
[154]	RF 900 MHz pulsed	0.0516–0.0054 W/kg	Long-term exposure	Mice	—	Alterations of electron potential of mitochondrial membrane DNA damage
[146]	RF 902.4 MHz	0.00516–0.0054 W/kg	Long-term exposure	Mice	—	Decreased antioxidant defences Lipid peroxidation DNA damage Decreased sperm count
[142]	RF 900 MHz	0.0067 W/kg	Long-term exposure	Rat	Constant	Imbalance in total antioxidant capacity Lipid peroxidation
[155]	RF 1800 MHz	0.15 W/kg	Short-term exposure	Mouse germ cells	—	Increased ROS production DNA fragmentation Oxidative DNA damage
[143]	RF 1800 MHz	4 W/kg	Short-term exposure	Mouse germ cells	—	Oxidative DNA damage Increased autophagy

**(b):** 

References	Radiation	Magnetic flux density	Exposure time	Model	Temperature	Effect
[159]	ELF-MF 50 Hz	1 mT	Short-term exposure	Boar	—	Lower mitochondrial activity
[160]	ELF-MF 50 Hz	8 mT	Long-term exposure	Rat	—	Mitochondrial damage
[161]	ELF-MF 50 Hz	5 mT	Short-term exposure	Human spermatozoa	Constant	Increased mitochondrial metabolism efficiency
[149]	ELF-MF 50 Hz	100 *μ*T	Long-term exposure	Rat	Constant	Decreased antioxidant defences Decreased sperm motility
[103]	ELF-MF 60 Hz	1 mT	Long-term exposure	Rat	—	Mitochondrial damage
[150]	ELF-MF 50 Hz	100–500 *μ*T	Long-term exposure	Rat	Constant	No on effect rat sperm count No effect on oxidative stress Increased caspase 3 activity
[109]	ELF-MF 50 Hz	500 *μ*T	Long-term exposure	Rat	—	No effect on oxidative stress
[147]	ELF-MF 120 Hz	2.5–8 mT	Short-term exposure	Mouse germ cells	—	Decreased viability Increased ROS production Increased apoptosis
[148]	ELF-MF 50 Hz	2.5 mT	Short-term exposure	Mouse germ cells	—	No effect on mitochondria Increased ROS production Decreased bcl-2 protein level
