# Corrigendum to “Role of Mitochondria in the Oxidative Stress Induced by Electromagnetic Fields: Focus on Reproductive Systems”

**DOI:** 10.1155/2020/5203105

**Published:** 2020-05-17

**Authors:** Silvano Junior Santini, Valeria Cordone, Stefano Falone, Mahmut Mijit, Carla Tatone, Fernanda Amicarelli, Giovanna Di Emidio

**Affiliations:** ^1^Dept. of Life, Health and Environmental Sciences, University of L'Aquila, 67100 L'Aquila, Italy; ^2^Infertility Service, San Salvatore Hospital, L'Aquila, 67100 L'Aquila, Italy; ^3^Institute of Translational Pharmacology (IFT), CNR, 67100 L'Aquila, Italy

In the article titled “Role of mitochondria in the oxidative stress induced by electromagnetic fields: focus on reproductive systems” [1], information was omitted in the “Acknowledgments.” The corrected section appears below:
